# Complete Genome Sequence and Characteristics of Mycobacteriophage IkeLoa

**DOI:** 10.1128/mra.00985-22

**Published:** 2022-10-26

**Authors:** Hannah R. Wheatley, Sarah R. Kushner, Frederick N. Baliraine

**Affiliations:** a Department of Biology & Kinesiology, LeTourneau University, Longview, Texas, USA; Portland State University

## Abstract

Mycobacteriophage IkeLoa is a lytic myovirus. It has a circularly permuted 155,280-bp genome containing 233 putative protein-coding genes, 32 tRNA genes, one tmRNA gene, and 64.7% G+C content. The RNA genes are distributed in five clusters across the genome. Only 28% of IkeLoa’s protein-coding genes can be assigned functions.

## ANNOUNCEMENT

Bacteriophages or “phages,” viruses that infect bacteria, were independently discovered by Frederick Twort and Felix d’Herelle (1–4). The bacterium-killing ability of phages inspired Felix d’Herelle to introduce the idea of phage therapy, but by the 1940s, phage therapy lost traction for various reasons, including the availability of effective and easier-to-administer antibiotics (5). However, the emergence of multidrug-resistant bacterial infections has renewed interest in phage therapy (1, 2, 6–8). Here, we report on the lytic bacteriophage IkeLoa.

**FIG 1:**
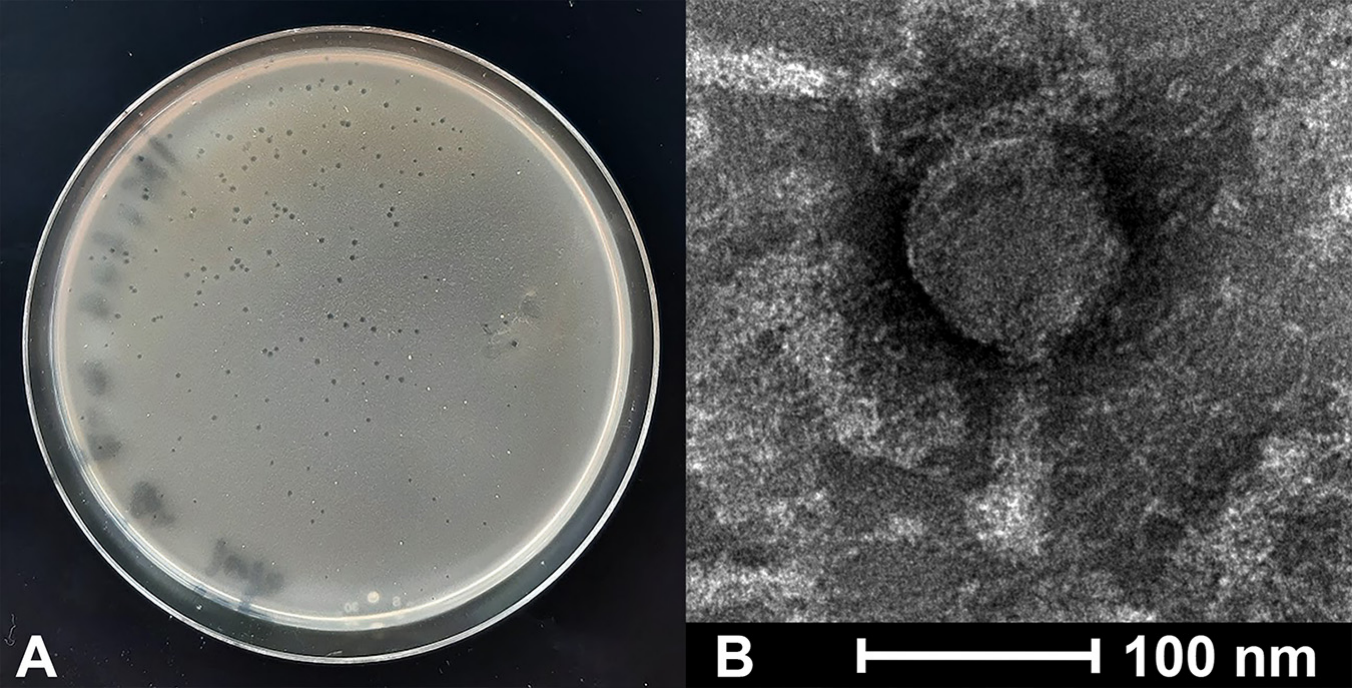
(A) Image of plaques formed by phage IkeLoa following a 48-hour incubation at 37°C in Middlebrook 7H9 top agar with M. smegmatis mc^2^155. (B) Transmission electron micrograph of phage IkeLoa. Phage particles were added to 300-mesh carbon-Formvar-coated copper grids (Ted Pella Inc., Redding, CA), negatively stained using 1% (wt/vol) uranyl acetate, and imaged at the Digital Microscopy Laboratory at the University of Arkansas for Medical Sciences.

Mycobacteriophage IkeLoa was isolated from a soil sample collected from a landscaping bush at LeTourneau University in Longview, TX (32.465224 N, 94.726598 W), on 2 September 2021 using standard methods (9). Briefly, the soil was washed in 7H9 liquid medium, and the wash was collected by centrifugation and filtration (0.22-μm pore size). The filtrate was inoculated with Mycobacterium smegmatis mc^2^155 and incubated with shaking at 25°C for 3.5 days. A filtered sample of the culture was plated in 7H9 top agar with M. smegmatis. IkeLoa, which produced clear plaques with an average diameter of ~0.7 mm (range, 0.5 to 0.8 mm) after 48 h at 37°C (Fig. 1A; *n* = 10), was purified through three rounds of plating. Negative-stain transmission electron microscopy showed IkeLoa to be a myovirus with an isometric capsid and a contractile tail, measuring ~100 nm (range, 94 to 106 nm) in diameter and length, respectively (Fig. 1B; *n* = 10).

Genomic DNA was extracted from a lysate of 2.8 × 10^11^ PFU/mL using the Promega Wizard DNA cleanup kit, prepared for sequencing using the NEB Ultra II Library kit, and sequenced using an Illumina MiSeq sequencer (v3 reagents). It yielded 245,512 single-end 150-bp reads that constituted ~233-fold shotgun coverage of the genome. Untrimmed reads were assembled and checked for completeness using Newbler v2.9 (10) and Consed v29 (11), as previously described (12). This revealed that IkeLoa has a circularly permuted, 155,280-bp-long genome with a similar G+C content to that of its isolation host M. smegmatis mc^2^155 at 64.7% (13).

IkeLoa was annotated using DNAMaster v5.23.6 (https://phagesdb.org/DNAMaster/), Glimmer v3.02 (14), GenMark v2.5p (15), HHpred (16, 17), ARAGORN v1.2.41 (18), tRNAscan-SE v2.0 (19, 20), BLAST in NCBI and PhagesDB (21, 22), TMHMM v.2.0 (23), SOSUI (24), Phamerator (25), and Starterator (http://phages.wustl.edu/starterator/). All programs were run using the default settings (26). Overall, 233 protein-coding genes, 32 tRNAs, and 1 tmRNA were predicted. The RNA genes were distributed in five distinct clusters across the genome, at bp positions 29800 to 125690. All but 4 genes in IkeLoa’s genome are on the sense strand. Using the gene content similarity (GCS) tool in PhagesDB (27), IkeLoa was assigned to subcluster C1 based on ≥35% GCS similarity to other phages in the database (28).

Functions could be assigned to only 28% (65/233) of the predicted genes, which included baseplate wedge protein and baseplate J protein, which play key roles in phage adsorption and infectivity (29), together with lysin A, holin, and lysin B, among other genes. No integrase, excise, and immunity repressor genes were identified, suggesting a lytic life cycle consistent with cluster C1 phages.

### Data availability.

Raw reads of mycobacteriophage IkeLoa are available in the Sequence Read Archive (SRA) database with accession no. SRX14483221, and its complete genome sequence is available in GenBank with accession no. OP021682.
